# Load Distribution on PET-G 3D Prints of Honeycomb Cellular Structures under Compression Load

**DOI:** 10.3390/polym13121983

**Published:** 2021-06-17

**Authors:** Olimpia Basurto-Vázquez, Elvia P. Sánchez-Rodríguez, Graham J. McShane, Dora I. Medina

**Affiliations:** 1 Tecnologico de Monterrey, School of Engineering and Science, Atizapan de Zaragoza 52926, Estado de Mexico, Mexico; olimpia.basurto@exatec.tec.mx (O.B.-V.); elvia.sanchez@tec.mx (E.P.S.-R.); 2 Department of Engineering, University of Cambridge, Trumpington Street, Cambridge CB2 1PZ, UK; gjm31@cam.ac.uk

**Keywords:** 3D-printing, additive manufacturing, PET-G, honeycomb cellular structure

## Abstract

Energy resulting from an impact is manifested through unwanted damage to objects or persons. New materials made of cellular structures have enhanced energy absorption (EA) capabilities. The hexagonal honeycomb is widely known for its space-filling capacity, structural stability, and high EA potential. Additive manufacturing (AM) technologies have been effectively useful in a vast range of applications. The evolution of these technologies has been studied continuously, with a focus on improving the mechanical and structural characteristics of three-dimensional (3D)-printed models to create complex quality parts that satisfy design and mechanical requirements. In this study, 3D honeycomb structures of novel material polyethylene terephthalate glycol (PET-G) were fabricated by the fused deposition modeling (FDM) method with different infill density values (30%, 70%, and 100%) and printing orientations (edge, flat, and upright). The effectiveness for EA of the design and the effect of the process parameters of infill density and layer printing orientation were investigated by performing in-plane compression tests, and the set of parameters that produced superior results for better EA was determined by analyzing the area under the curve and the welding between the filament layers in the printed object via FDM. The results showed that the printing parameters implemented in this study considerably affected the mechanical properties of the 3D-printed PET-G honeycomb structure. The structure with the upright printing direction and 100% infill density exhibited an extension to delamination and fragmentation, thus, a desirable performance with a long plateau region in the load–displacement curve and major absorption of energy.

## 1. Introduction

In a dynamic world, collisions between two or more objects are inevitable in everyday life. Among the physical phenomena that can be generated by the kinetic energy of an impact are perforation, spallation, fracture, and fragmentation, which cause adverse effects on the objects or persons involved [1,2]. Therefore, safety systems and energy absorbers should be enhanced for protection purposes and improvement of crashworthiness; reducing injuries to the involved moving parts in a wide range of engineering applications (e.g., civil, mining, aerospace, automotive and transportation, and biomedical) [3].

Several studies [4,5,6,7,8] reported cellular structures, which consist of periodically repeated and connected elementary cells [9], as internal architectures for the development of new materials with enhanced energy absorption (EA) capabilities. Among the various designs of cellular structures, the hexagonal honeycomb is widely known and intensively studied owing to its space-filling capacity, structural stability, and high EA potential [8,9,10,11]. Nevertheless, there is a need to make previous structures conform to complex geometries [8], which are related to the manufacturing process. Therefore, in recent years, there has been a growing interest in the development of additive manufacturing (AM) processes in energy-absorbing structures and deformation mechanisms [9,10].

AM (i.e., three-dimensional [3D] printing) is a processing technique for creating 3D objects by adding two-dimensional layers of material, one layer at a time. This method enables the formation of complex geometries and a selection of materials from preformed processible materials [12]. Fused deposition modeling (FDM) is the most popular AM method. In this process, a 3D printer extrudes the filament material through a small nozzle and via a heated extruder, and it deposits fine layers upon each other to create the desired object [13].

Material selection is essential in AM, especially for engineered structural applications. In FDM 3D printing, most of the polymers used are amorphous thermoplastic filaments [14]. These include acrylonitrile–butadiene–styrene (ABS) copolymers, polycarbonates (PC) [15], and semicrystalline thermoplastics with low crystal grade (e.g., polylactic acid [PLA] [16] and polycaprolactone [PCL] [8,17]).

Among the mentioned materials, the most widely used are PLA, ABS, and, recently, polyethylene terephthalate glycol (PET-G) [18,19].

PET-G, an amorphous copolymer, is a novel material on the market [20,21,22]. PET-G polymer is ductile, durable, highly chemically and impact-resistant, biocompatible, tough, flexible, readily thermoformable [22,23], ultraviolet- and weather-resistant [24,25,26], and easier to process and mold than polyethylene terephthalate (PET) [21,27]. In comparison with other 3D printing filaments, PET-G is considered a material that possesses the qualities of ABS such as durability and strength but is biocompatible and has higher flexibility. At the same time, PET-G has the ease of printing, biocompatibility, and recyclability like PLA [19]. Table 1 provides key material characteristics of ABS, PLA, and PET-G. In this way, PET-G becomes an excellent option for the demand for 3D-printed polymeric parts in innovation-driven applied fields such as robotics, automotive, aerospace, and bioengineering [10].

Previous studies on the mechanical properties of PET-G structures processed by FDM [25,26,31,32,33] analyzed the material by performing an experimental investigation on tensile properties. However, little work has been performed to evaluate the crashworthiness behavior of PET-G honeycomb structures due to the manufacturing and machine-processing related factors from FDM printing.

The FDM process involves several physical phenomena, such as fluid flow, heat transfer, solidification, and filament bonding [34]. The key to producing a mechanically strong 3D-printed part is the interdiffusion and re-entanglement of the heated extruded thermoplastic across the bonding interfaces [35]. Therefore, the layered manufactured effect of the FDM process has a significant impact on the bulk composition and surface. Even though it is not possible to fully eliminate the layer manufacturing effect of the process, efforts such as the optimization of process parameters can be employed [36]. Characterization of manufactured structures should be given sufficient attention in using 3D-printed parts for engineering purposes. The literature reveals [37,38,39] that FDM input parameters, involved in the processing conditions, such as layer thickness, printing angle of layers, build direction, infill pattern, and infill density, are crucial for improving layer adhesion and surface roughness [36,37,40].

In this study, 3D honeycomb structures of PET-G were prepared using FDM.

Regarding the manufacturing of the test specimens, this study mainly focuses on varying two process parameters of the 3D printer: infill density and building direction. Meanwhile, based on studies by Rodríguez-Panes et al. [41], Durgashyam et al. [31], Dupaix and Boyce [33], and Srinivasan et al. [25], the layer thickness, infill pattern, and printing angle of layers remained constant to evaluate the mechanical and surface properties of FDM printed PET-G parts as a means of gaining better insight into the relations between the 3D printed morphology of the honeycomb structure and the mechanical performance as the infill density and building direction are changed. The response of the hexagonal honeycomb was analyzed quasi-statically via compression essays along the transverse direction from the XY plane to determine the effects of the selected mechanical properties on the performance of the structure.

## 2. Materials and Methods

The printing system used was a Zortrax M200 Plus. The machine has an extruder head with a 0.4-mm-diameter nozzle and positioning accuracy of 1.5 μm in the XY plane and 1.25 μm in its Z axis.

**Figure 1:**
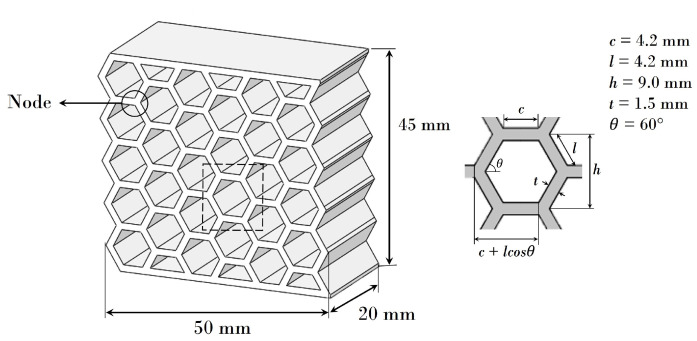
Geometry and parameters of the honeycomb structure.

The material for the manufacture of the specimens was a thermoplastic PET-G filament supplied by Zortrax. The diameter of the filament was 1.75 mm, and it was provided in pools of 800 g. The 3D honeycomb structure was designed on SolidWorks 2019 (Dassault Systèmes SolidWorks Corporation, Waltham, MA, USA). The honeycomb specimens were manufactured with dimensions of 5 × 4.5 × 2 cm^3^; they consisted of 5 × 6 hexagonal cells and had a cell width/wall thickness ratio of 5.6 (Figure 1).

**Figure 2:**
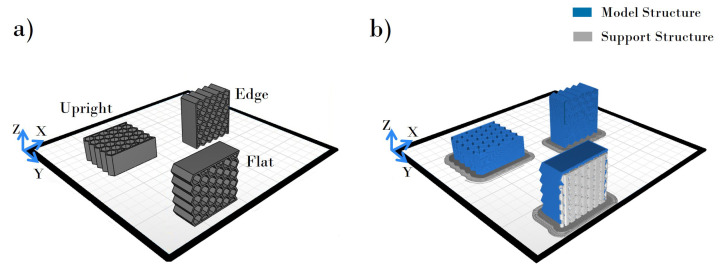
(**a**) Honeycomb structure at different orientations for 3D printing. (**b**) Generated support for different printing orientations.

The assumptions underlying the test specimens depended on the variation of two preprocessing parameters with three levels, namely, infill density (30%, 70%, and 100%) and printing orientation of the model (edge, flat, and upright), which were considered controlling factors. The build direction was modified by changing the orientation of the model and thus the direction of layers (Figure 2).

For reference purposes and for checking the repeatability of the experimental results, three specimens of PET-G 3D prints of honeycomb cellular structures were printed and tested one by one using the FDM processing configurations presented in Table 2. The series code is made from the letter H, for honeycomb structure, followed by the first letter from the type of printing orientation (e.g., edge, flat, or upright) and the value for the infill density parameter (e.g., 30%, 70%, or 100%). The test number can be identified after a hyphen following the infill density value. Table 3 shows the fixed configuration used in each print.

The morphology of the 3D-printed PET-G samples was imaged using a scanning electron microscope (JEOL-JSM-6360 LV) working in high-vacuum mode with an accelerating voltage of 20 kV, varied magnifications, and a working distance of 13 mm.

Uniaxial strain compression essays were performed, at room temperature, by using an INSTRON model DX static hydraulic universal testing system (UTM 600DX-F2-G1), with a capacity of 600 kN and closed-front crossheads. It is equipped with a bottom plate that moves upwards, applying a compressive force to the sample via a compressive plate until the specimen was compressed to 50% relative to the transverse direction from the XY plane. The speed of compression was configured to be constant at 1.0 mm/min for all the essays. The deformation behavior of the specimens was video recorded.

## 3. Results and Discussion

### 3.1. Phase Morphologies

**Figure 3:**
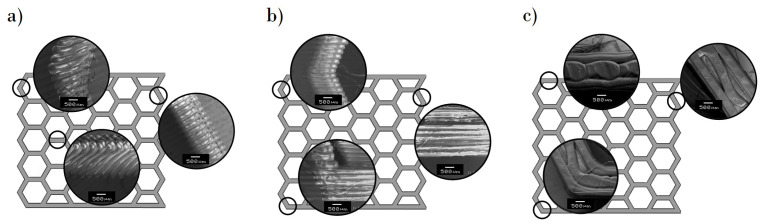
Filament print path through hexagonal honeycomb structure at 100% infill density of (**a**) edge orientation printing, (**b**) flat orientation printing, and (**c**) upright orientation printing.

**Figure 4:**
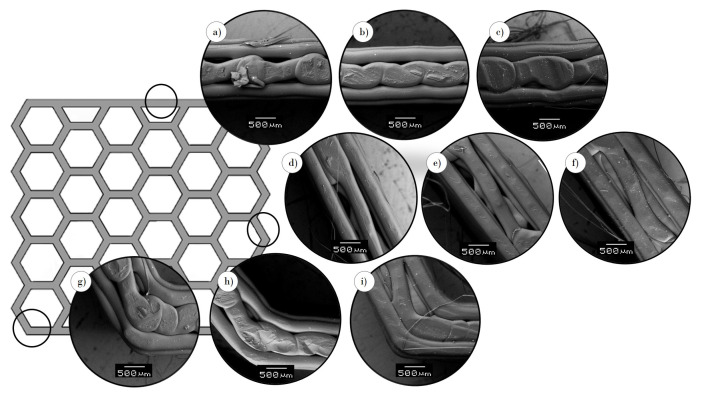
Microscopic characterization of upright printed structure: front superior wall of density at (**a**) 30%, (**b**) 70%, and (**c**) 100%; front lateral wall at (**d**) 30%, (**e**) 70%, and (**f**) 100%; front corner wall at (**g**) 30%, (**h**) 70%, and (**i**) 100%.

The microscopic characterization shows the raster of the 3D-printed structures. Through an analysis of Figure 3 and Figure 4, the direction of the filament and the quality of the structures were verified, respectively.

The build direction was essential for both the edge (Figure 3a) and flat (Figure 3b) printing direction because the lateral walls of the specimens were composed of horizontal parallel fused beads, which are perpendicular to the compression force. For the upright-printing-direction specimens (Figure 3c), the lateral walls had vertical parallel fused beads that are parallel to the applied force. Therefore, the printed part exhibited anisotropy, and its mechanical properties depended on the orientation of the filament, which accorded with the application of the layers one onto another in the Z axis. When the layers are parallel to the compression force, the sample exhibits high flexural strength; however, the sample showed weakness in the walls for layers that were perpendicular to the applied force. In the case of the upright printing direction, the horizontal walls were formed by crossed lines (Figure 4).

The smaller the infill density of the structure, the smaller the relative density and the smaller the amount of material used to manufacture it. Moreover, the welding of the filament is affected by air gaps. An air gap is defined as the space between the road of the filaments; the air gap is zero if the filaments are in perfect contact; it is positive if the pathways of the filaments do not touch each other (Figure 4a,d,g); a negative air gap means the filaments are overlapping (Figure 4c,f,i) [37]. Figure 4 shows that the value of the air gaps depended on the infill density of the sample. With an increase in the infill density, the air gap became negative, and there was better welding between adjacent filaments; i.e., specimens with 100% infill density had negative air gaps, which decreased the possibility of brittle fracture or delamination.

### 3.2. Axial Crushing Behavior

The response of the hexagonal honeycomb was analyzed quasi-statically via compression essays along the transverse direction from the XY plane to determine the effects of the selected mechanical properties on the performance of the structure.

**Figure 5:**
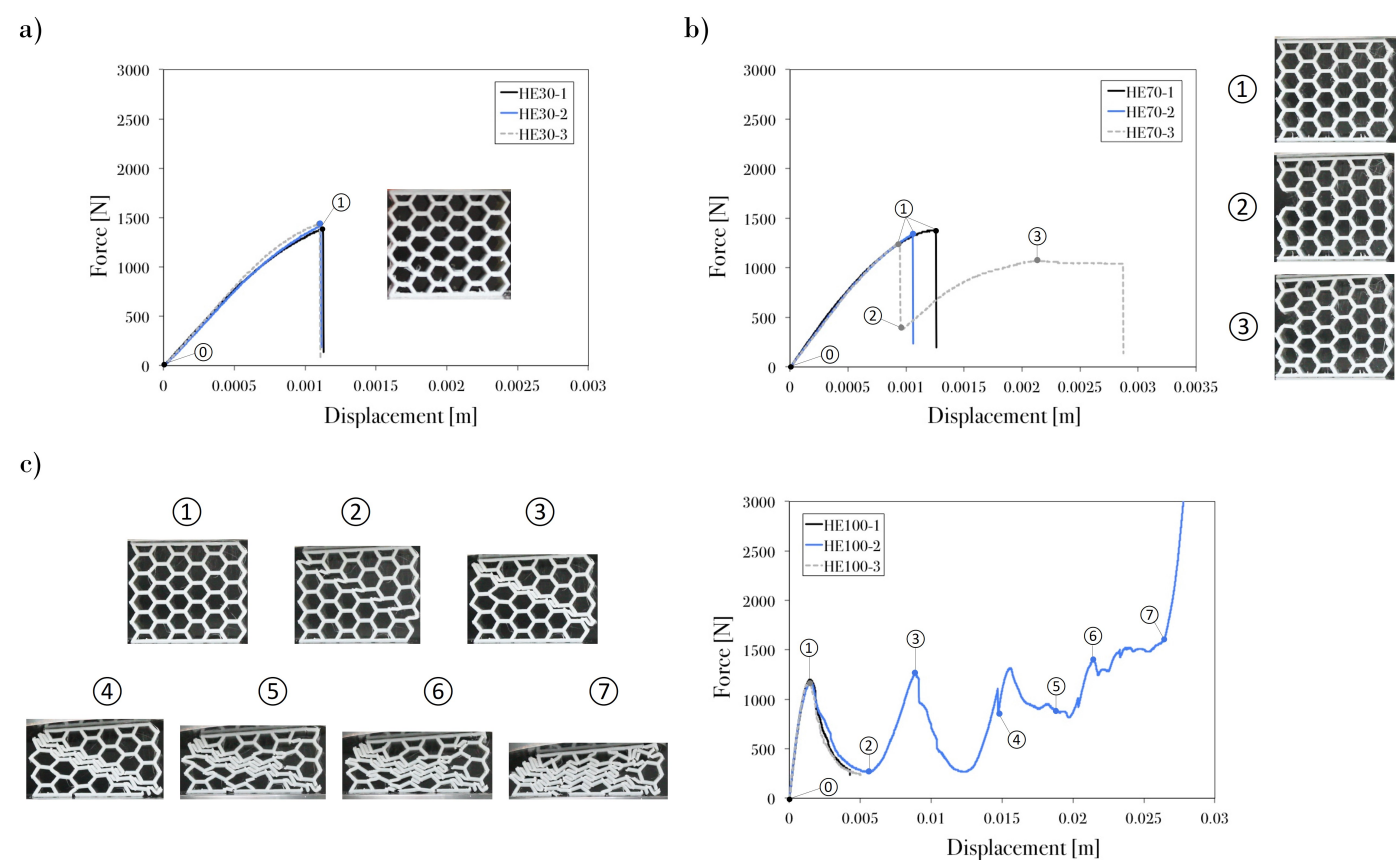
Displacement response of PET-G honeycomb specimen at edge orientation printing and infill density of (**a**) 30%, (**b**) 70%, and (**c**) 100%.

**Figure 6:**
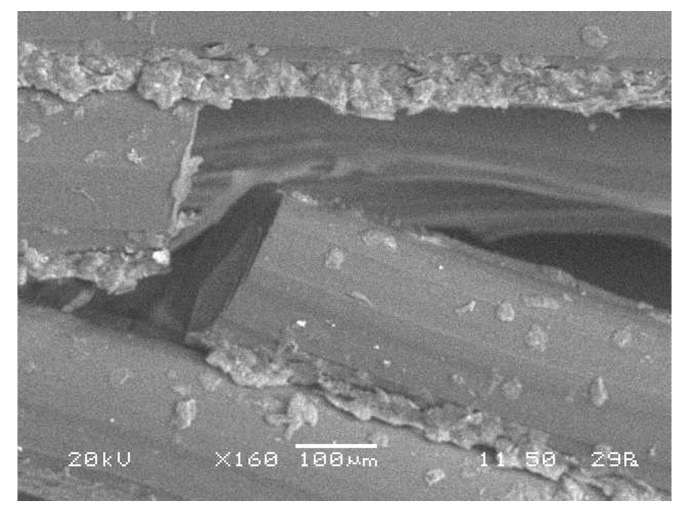
SEM micrograph of failure microstructure of PET-G HE100-2 honeycomb structure.

**Figure 7:**
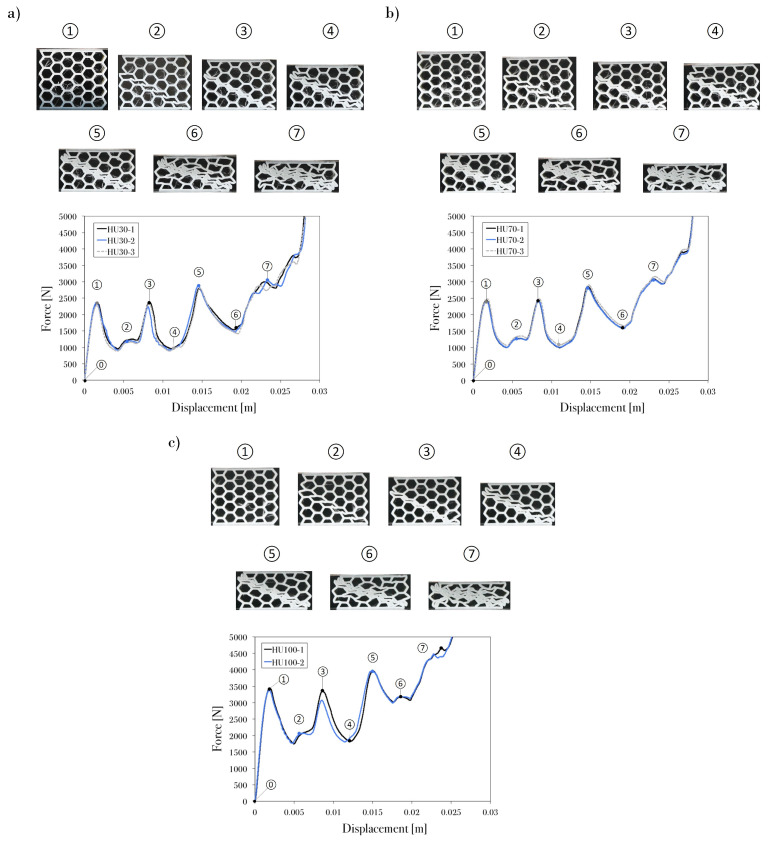
Crushing sequence and displacement response of the PET-G honeycomb at upright orientation printing and infill density of (**a**) 30%, (**b**) 70%, and (**c**) 100%.

**Figure 8:**
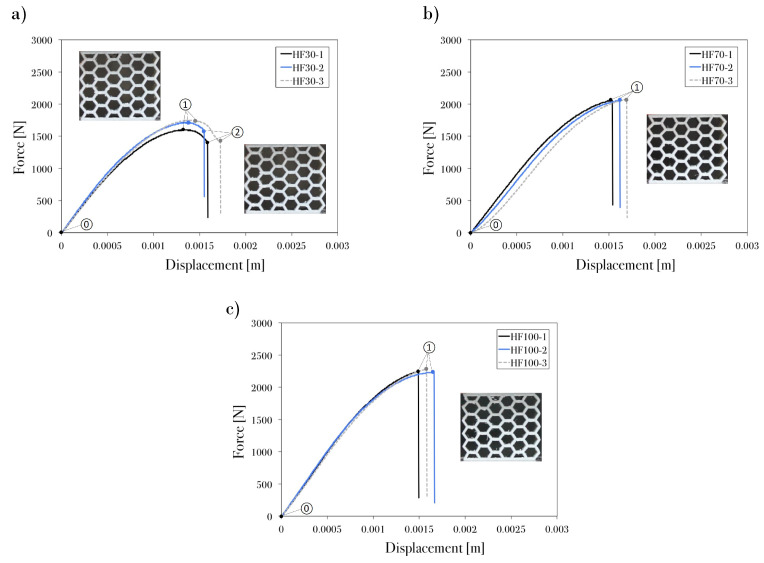
Displacement response of PET-G honeycomb specimen at flat orientation printing and infill density of (**a**) 30%, (**b**) 70%, and (**c**) 100%.

The following figures (Figure 5, Figure 6, Figure 7 and Figure 8) show the behavior of the PET-G honeycomb structures during the compression tests. The figures depict the sequence of deformed configuration and the recorded load–displacement response from the three samples of each specimen. The tests ended automatically by the machine after detecting a break with a high rate of load fall or ended by the user after the densification region was reached. Previous studies show two types of fractures: ductile and brittle. The main characterization of ductile fracture is the presence of large plastic deformations that occur before and/or during the fracture process; in brittle fracture, there is a small amount of inelastic deformation [42]. In engineering applications, ductile fracture is less damaging because it occurs over a period of time, whereas brittle fracture is fast, unpredictable, and often catastrophic.

The specimens printed with the edge printing direction and infill density of 100% (Figure 5c) exhibited a staircase effect. Brittle fractures were found between the layers, which were caused by the low resistance of the filaments. The reason for the low resistance was that the orientation was normal to the applied force and the layers rearranged with each microfracture of the structure. Even though brittle fractures weakened the structure, the improved welding and separation of layers (Figure 6) provided an extension to the plateau region. The peaks and valleys in this section of the curve correspond to the fracture of individual cell walls. Progressive, folding, and hinging deformations were observed. Two of the three tests with HE100 configuration (Figure 5c), were automatically finished by the compression machine after detecting a high fall load following the initial peak load (Point 1) from an unexpected brittle fracture on the structure. Nevertheless, it can be observed that the specimen’s performances at the first peak (Point 1) and valley (Point 2) are consistent. Therefore, a progressive folding deformation could be expected with the HE100 configuration.

For the specimens in the upright printing direction, cracking could be explained as the delamination of the infill raster (Figure 7). Previous studies [9,10,43] suggest that, in FDM-3D-printed structures, the delamination of layers affects the material behavior. Force–displacement curves are highly similar for this printing orientation regardless of the infill density. Nevertheless, in this study, the infill density of 100% (Figure 7c) had a better response due to the higher welding quality. Delamination and ductile microfractures occurred in this printing direction because of the layer orientation and infill density (air gaps) of the filaments (Figure 4), which partly explained the low welding quality in the low-infill-density specimens (Figure 7a,b).

In the flat-printing-direction specimens (Figure 8), support material was built during the printing process [44] because of the printing orientation. Therefore, the model preserved burrs (i.e., projections of unwanted material beyond the desired manufactured features) in its cells, which changed the processing conditions from the optimal ones and resulted in a ductile–brittle transition [45]. The specimens with infill density values of 70% and 100% presented spontaneous brittle fracture (Figure 8b,c); this is related to the orientation of the layers and the high concentration of the filaments. Meanwhile, the axial behavior of the infill density of 30% (Figure 8a) showed ductile fracture before the complete breakage of the structure. Nevertheless, it had a smaller peak crushing force than those samples with 70% and 100% infill density.

In general, the load–displacement curves of the honeycomb groups exhibited very similar behaviors. Specimens HE30, HE70, HF30, HF70, and HF100 presented linear elastic deformations, which ended once the critical stress was reached and brittle fractures and plastic yielding were observed. HE100, HU30, HU70, and HU100 presented three phases (linear elasticity, plateau region, and densification) in their load–displacement curves; the hexagonal structures exhibited the high-repeatability collapse of the diagonal array in the plateau region, where fracture started to occur plastically at the joints that connected the cell walls. Therefore, aside from the cell failure that governs the plateau stress, the load distribution in each printed layer and the welding strength had a significant effect on the honeycomb performance.

A correlation can be observed between the fracture mode and the loads from the compression test. According to the classification of Mamalis et al. [46] and Silva et al. [47], the main fracture modes that were identified in this study were as follows: (I) brittle fracture with progressive crushing, folding and hinging; (II) brittle fracture with catastrophic failure; and (III) ductile fracture with progressive folding. Table 4 shows the collapse mode observed in each specimen.

Specimens HE30, HE70, HF30, HF70, and HF100 presented fracture mode II. This mode is related to the unstable and catastrophic failure of the structure, which occurred as parts of the model broke and exhibited unpredictable kinetic dissipation that depended on the location of weak welding in the layers. Meanwhile, mode I was observed in the HE100 sample; the brittle fracture was formed at the early stage and along the plateau region of the crushing event, thus decreasing the stability of the structure. The upright-printing-direction structures showed mode III fracture; each sample exhibited a localized brittle fracture that was generally ductile, which allowed the material to fail progressively without affecting the stability of the structure. Thus, there was no unpredictable and destructive failure.

The behavior of the material is another factor to be considered. Before the heat treatment, the polymeric material used as the filament for FDM 3D printing had high plasticity; after printing, with an increase in stiffness, the maximum strain decreased [9]. For EA applications, the most relevant properties were the plateau stress and densification strain. A desirable characteristic of cellular materials is a long plateau region in the load–displacement curve [9], which was observed in specimen HU100 (Figure 7c). For honeycomb structures with a low infill density and a printing orientation different from the upright direction, the plastic collapse of the walls is the mechanism that governs the plateau stress. Meanwhile, the plateau stress is governed by the zigzag deformation mechanism for specimens with the upright direction.

### 3.3. Crashworthiness Criteria

For a full understanding of the EA performance of hexagonal honeycomb structures under compressive force, multiple indicators can be adopted, such as energy absorption (EA), specific energy absorption (SEA), peak crash force (PCF), mean crash force (MCF), and crash force efficiency (CFE), which are defined by the load–displacement curve and can be obtained by calculating the area below the curve of the plateau region [48,49,50,51,52].

The EA of structure describes its capacity to dissipate impact energy, and it directly correlates with the impact force as a function of displacement *F(x)* and the effective crushing deformation *d*, which is the length of the plateau stage. EA can be written mathematically as follows:

(1) $$EA=\int_{0}^{d}F\left(x\right)dx$$

The SEA is the energy absorbed per unit mass under compression.

(2) $$SEA=\frac{EA}{m}$$

where *m* is the mass of the structure. High SEA values are preferred because they result in higher energy absorption efficiency of structures [49].

PCF and CFE are critical parameters from the perspective of energy absorption stability. PCF can be directly obtained from the curve. MCF represents the average force during the compressive test [49,50].

The MCF can be obtained by using the following expression:

(3) $$MCF=\frac{1}{d}\int_{0}^{d}F\left(x\right)dx=\frac{EA}{d}$$

The CFE is the ratio of the MCF to the PCF and can be calculated as follows:

(4) $$CFE=\frac{MCF}{PCF}$$

The displacement point at which the structure starts the densification is calculated by finding the energy efficiency *f*, which can be defined as [53]:

(5) $$f=\frac{EA}{F_{max}}$$

where $F_{max}$ is the maximum crush force in the crushing distance. Equation (5) renders the maximum value where densification starts, and the corresponding displacement value, effective stroke $S_{ef}$, is divided by the total length of the structure under compression to obtain the percentage of densification.

The crashworthiness indicators are obtained by identifying the area under the plateau region curve of the performed compression tests that presented a plateau region, by referring to the densification phase, and by using Equations (1)–(5). These indicators are summarized in Table 5.

**Figure 9:**
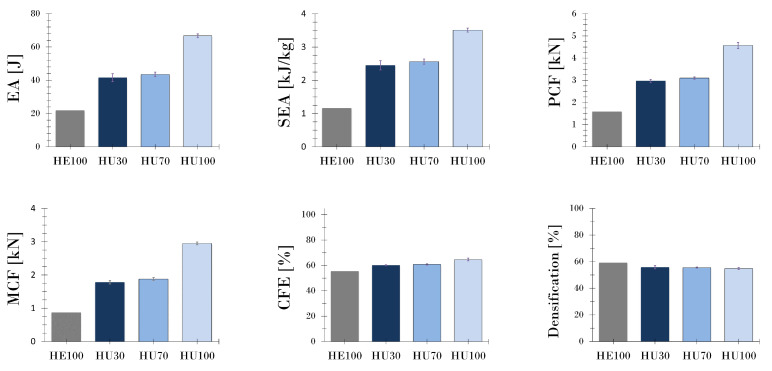
Comparison of the crash performance parameters.

Regarding the EA characteristics with the failure mode and 3D printing parameters, observation showed that the upright orientation of printing provided a collapse mode in which the material progressively failed without instabilities (Figure 9). In the HU samples, the EA, SEA, and PCF values increased with the infill density. The layer orientation of the edge- and flat-printing-orientation specimens was perpendicular to the compressive force, which caused higher stress concentrations along the filaments and produced a brittle fracture. The specimens with edge and flat printing directions and low infill density values exhibited layer separation, which caused instabilities and structural weakening. In the specimens with high infill density values, the filaments were exposed to a compressive force, which could cause a spontaneous catastrophic failure along the filaments. Therefore, even though the PCF is greater at higher infill density values, EA directly affects the compressive parameters (e.g., length of compression and MCF) and the long plateau region, which in the FDM method is also correlated to the infill density and printing orientation.

In general, the results showed that the zigzag mechanism of failure provided a longer plateau region owing to the progressive deformation of the printed structures, which, along with delamination, was caused by the layer-upon-layer method of 3D printing. Features such as irreversible energy conversion, long stroke (plateau region), stable and repeatable deformation mode, lightweight and high SEA capacity, and cost-effectiveness are part of the fundamental principles for designing EA, where the purpose is to dissipate kinetic energy in a controlled mode or at a predetermined rate [54]. There was no significant difference between the EA and SEA, given that the mass did not have a notable effect because of the structure size. However, at a higher infill density, a structure has more mass.

## 4. Conclusions

In the 3D printing process, multiple processing parameters impact the mechanical and superficial properties of the manufactured part. The implementation of this manufacturing technology in energy-absorbing structures requires a proper characterization because the actual material properties depend on the printing processing conditions and design model. The area under the curve from each compression essay is an indirect method of obtaining a structure’s absorbed energy. In this work, the crushing resistance and EA performance of PET-G honeycomb structures were comprehensively investigated.

From the FDM AM, the presence of air gaps in the printed structure and the stress concentration along the filament beads affected the results by causing cracks. The process parameters in FDM and mechanical anisotropy directly affect the fracture modes and EA capabilities of the structure. A periodic cellular structure fails in a progressive manner. Therefore, with correct parameter configuration, the layer-by-layer welding property of the FDM method provides an extension to delamination and fragmentation, and spontaneous catastrophic failures could be avoided. Thus, the structure can absorb and dissipate higher energy by transforming it into internal energy via deformation work.

The test results suggest that the printing parameters implemented in this study considerably affected the compressive mechanical properties of the 3D-printed PET-G honeycomb structure. Overall, the best results were obtained with 100% infill density and the upright printing direction. Therefore, it contrasts with the optimized building positions for high tensile strength and elongation of edge and flat printing direction [32,33] but coincides with having the layers parallel to the applied force.

## Figures and Tables

**Table 1:** Properties of FDM filaments.

Material	Characteristics	Extrusion Temp (**°C**)	Elastic Modulus (GPa)	Refs.
ABS	Strong and durable, good temperature resistance, susceptible to warping. Petroleum-based plastic.	230–250	2.3	[28,29,30]
PLA	Biopolymer with biocompatibility, low impact strength, and temperature resistance.	180–210	3.8	[28,29,30]
PET-G	Durable, biocompatible, recyclable, tough, high impact and chemical resistance.	210–245	3.0	[28,29,30]

**Table 2:** Descriptions and specifications of case studies.

Series Code	Orientation	Infill Density (%)
HE30	Edge	30
HE70	Edge	70
HE100	Edge	100
HF30	Flat	30
HF70	Flat	70
HF100	Flat	100
HU30	Upright	30
HU70	Upright	70
HU100	Upright	100

**Table 3:** Fixed preprocessing parameters.

Layer thickness	0.19 mm
Raster angle	45°
Platform temperature	30°
Infill pattern	Honeycomb
Support	Lite and Smart bridges

**Table 4:** Fracture mode of test specimens under quasi-static compression.

Series Code	Collapse Mode
HE30	II
HE70	II
HE100	I
HF30	II
HF70	II
HF100	II
HU30	III
HU70	III
HU100	III

**Table 5:** Crashworthiness indicators of test specimens under quasi-static compression that presented a plateau region after FDM.

Series Code	PCF [kN]	EA [J]	SEA [kJ/kg]	MCF [kN]	CFE [%]	Densification [%]
HE100	1.58	21.80	1.16	0.87	55.41	59.11
HU30	2.97 ± 0.07	41.57 ± 2.37	2.45 ± 0.14	1.78 ± 0.05	60.08 ± 0.42	55.79 ± 1.22
HU70	3.10 ± 0.05	43.50 ± 1.37	2.56 ± 0.08	1.88 ± 0.04	60.76 ± 0.43	55.52 ± 0.40
HU100	4.57 ± 0.14	66.71 ± 1.19	3.51 ± 0.06	2.95 ± 0.04	64.63 ± 1.04	54.86 ± 0.75

PCF, peak crash force; EA, energy absorption; SEA, specific energy absorption; MCF, mean crash force and CFE, crash force efficiency.

## Data Availability

All data, belonging to this work, is given and presented herein.

## Abbreviations

The following are used in this manuscript:

3D	Three Dimensional
AM	Additive Manufacturing
CFE	Crash Force Efficiency
EA	Energy Absorption
FDM	Fused Deposition Modeling
MCF	Mean Crash Force
PCF	Peak Crash Force
PET-G	Polyethylene terephthalate
PLA	Polylactic acid
SEA	Specific Energy Absorption
